# Is There a Cognitive Footprint of Political Systems? The Case of Separation and Reunification of East and West Germany and Its Association With Later Life Cognitive Health

**DOI:** 10.1177/01640275251383810

**Published:** 2025-10-04

**Authors:** Ariane Bertogg, Jeffrey E. Stokes

**Affiliations:** 1 Department of History, Sociology, Sports Science and Empirical Educational Research, University of Konstanz, Konstanz, Germany; 2 Department of Gerontology, University of Massachusetts Boston, Boston, MA, USA

**Keywords:** aging, cognitive functioning, political systems, socialization, East and West Germany

## Abstract

Germany’s division into the GDR (East) and FRG (West) in 1946, and subsequent reunification in 1989, had lasting impacts on individual life courses. While East-West differentials in physical health and mortality have been well-studied, cognitive health differences remain underexplored. This study examines cognitive trajectories among individuals living in the GDR and FRG, focusing on exposure duration and age at reunification. We find that former GDR residents perform better in memory, verbal fluency, numeracy, and orientation. Cognitive advantages for East Germans are most pronounced among individuals who experienced reunification in their twenties, suggesting a critical period during young adulthood. In contrast, West Germans who experienced reunification in their forties show relative disadvantages. No significant differences are observed among those near retirement age at the time of reunification. These findings contribute to understanding how political regimes shape cognitive aging and highlight the long-term effects of sociopolitical contexts on aging processes.

## Introduction

The separation of post-war Germany into an Eastern part (the German Democratic Republic, short: GDR) and a Western part (the Federal Republic of Germany, short: FRG) in 1946 and the subsequent reunification in November 1989 has had lasting consequences for the political landscape in Germany, Europe, and globally. Moreover, it has had lasting consequences for individuals’ life course experiences with regard to educational attainment, occupation and careers, family formation and the division of labor, and the formation of their personal and political values (Blossfeld, 2018; Gebel & Giesecke, 2009; Hanson & Wells-Dang, 2006; Zoch, 2020). One decade after reunification, East German men still exhibited lower life expectancy than West German men (Heinemann et al., 1996), while for women, life expectancy converged (Grigoriev & Pechholdová, 2017). These patterns are in line with comparisons of capitalist and post-socialist states (Abuladze et al., 2023; Ahrenfeldt et al., 2019; Formanek et al., 2019). Health behaviors – particularly smoking, drinking, and exercise – account for these differences, affecting cardiovascular health in particular (Heinemann et al., 1996). With regard to mental health, East-West differences in Germany are less clear (Otten et al., 2024; Schmalbach et al., 2023), suggesting gender- and outcome-specific patterns.

As concerns cognitive outcomes in later life, less research exists despite the observation that policies may leave a “cognitive footprint” (Rossor & Knapp, 2015). Based on non-representative insurance data, dementia prevalence was shown to be higher overall in East German regions (Kreft & Doblhammer, 2022; Teipel et al., 2015). Using a comparative approach including post-socialist and capitalist countries in Europe, Abuladze et al. (2023) find that older people in Eastern Europe report slightly better outcomes in cognitive functioning. Explanations for the latter finding include accumulated cognitive skills resulting from availability and use of early childcare (Ghirardi et al., 2023), occupational differences in job and workplace characteristics (Hülür et al., 2020), as well as context-specific returns on education that vary by age at reunification (Klein et al., 2019; Sander, 2014). Given that not all cognitive decline leads to dementia (Stern, 2002), these findings do not necessarily contradict each other but may be explained by heterogeneity in trajectories across political systems. Our study offers a new perspective, focusing on representative data and comparing older adults aged 50-90 born in East and West Germany before re-unification. More specifically, our study asks:

(1) Whether and how individuals who were socialized or lived in East or West Germany differ with regard to their levels and trajectories of cognitive health in later life.
(2) How these differences between regimes vary by duration of exposure to the system and, hence, by age at reunification.

In answering these questions, this study contributes to the literature in several ways.

First, it adds to the growing literature studying the *impact of welfare regimes* – including their political systems and health-relevant policies – on later life health in general. While a large part of the existing comparative research is focused on liberal democracies (or capitalist economies), countries that are under semi-democratic or authoritarian rule (and coordinated market economies) too face challenges of population aging. Our study provides a direct comparison of culturally similar and geographically neighboring national contexts – one capitalist, the other socialist – *to isolate the impact of the political system*.

Therefore, this study proposes a *methodologically refined identification strategy,* by making use of the German case as *a quasi-natural experiment,* due to the joint history before separation, and the re-unification. Thus, we also explore the nuanced impact of the political system by theorizing and operationalizing how the age at reunification – and thereby: how the duration of socialization – may moderate the impact of the political and educational system on later life cognitive health. Crucially, we are able to examine both *critical period* and *length of exposure/cumulative (dis)advantage* as potential explanations of East-West differences due to this unique study design.

Third, this study extends the existing literature documenting early mortality and physical health disadvantages for residents of post-socialist countries, yet ambiguous differences with regard to mental health. By focusing on cognitive health, which is affected by both physical health (particularly: cardiovascular and metabolic health) and mental health (particularly: loneliness, stress, and depression; Livingston et al., 2020), it adds a new dimension to the growing literature that studies the “long arm” of exposure to socialist rule. Cognitive functioning is a particularly suitable outcome, as its determinants span the entire lifespan, with educational exposure in early life playing a pivotal role in building cognitive reserve (Stern, 2002), and delaying the onset of dementia (Lövdén et al., 2020).

Finally, answering calls for studying the “cognitive footprint” of policies (Rossor & Knapp, 2015), we contribute to a better understanding of the social factors behind cognitive aging by examining a contextual feature that has to date been neglected. The relevance of our study is underscored by global trends of increasing life expectancy and rising prevalence of dementia, as one leading cause of death worldwide (Nichols et al., 2022). Despite recent advancements in pharmaceutical therapies, preventive efforts remains key to reducing the development and incidence of dementias (Livingston et al., 2020) and reducing their burden for individuals and their families (Connors et al., 2020; Wolters et al., 2019), public health systems (Kelley et al., 2015) and economies (Cantarero-Prieto et al., 2019; Mattap et al., 2022).

## Literature Review and Theoretical Considerations

### Welfare Regimes and Health

Studying differences between political regimes in Europe, it has been found that in Southern and Eastern European welfare states physical health is worse overall than in Scandinavian or West European welfare states (Eikemo et al., 2008; Mackenbach, 2017). This partly extends to cognitive functioning: Older adults’ cognitive functioning is poorer in Eastern Europe than in Northern or Western Europe, but better than in Southern Europe (Ahrenfeldt et al., 2019; Formanek et al., 2019). Contextual factors that have been positively associated with cognitive functioning are economic well-being (Leist et al., 2014) and gender-egalitarianism (Bertogg & Leist, 2023; Bonsang et al., 2017), but the cognitive footprint of political regimes has rarely been studied.

As concerns the physical health impact of welfare regimes specifically, it was found that social-democratic regimes host healthier residents (Muntaner et al., 2011), which can be attributed to egalitarian policies and the presence of a liberal democracy. The evidence of a positive impact of social-democratic regimes is less conclusive when it comes to the question of which welfare regimes do best at reducing health *inequalities* within their populations (Mackenbach, 2017; Muntaner et al., 2011). Post-socialist countries represent a heterogeneous group which should be studied separately (Eikemo et al., 2008). The present study thus focuses on the comparison between East and West Germany, which allows isolating the potential impact of *political* differences while cultural and demographic characteristics remain similar.

### Health Disparities Between East and West Germany: Mechanisms and Hypotheses

Given the strong link between *physical health* and cognitive functioning in later life (Qiu & Fratiglioni, 2015), and the clear physical health disadvantage among East Germans as compared to West Germans (particularly among men), observed even decades after re-unification (Heinemann et al., 1996; Luy et al., 2021; Mielck, 2000), these studies might suggest that East Germans should exhibit poorer cognitive functioning than West Germans. Another argument that supports the assumption of a cognitive disadvantage among East Germans is *economic development* (Leist et al., 2014). Market-driven economies, such as West Germany, exhibited faster economic growth after the second world war, creating opportunities for wealth accumulation and higher living standards (e.g., Staub, 2014). Similarly, the re-unification itself posed an economic shock for the East German population. Indeed, the early experience of economic stress among East Germans has been linked to lower metabolic health than among West Germans of the same birth cohort (Bister et al., 2022). Finally, marked differences in *nutrition* were observed between East and West Germany (Mensink & Beitz, 2004), persisting in some parts beyond the re-unification. Given the existing evidence on poor nutrition and lower cognitive functioning (Ampaabeng & Tan, 2013; Roseboom et al., 2011), individuals who grew up in East Germany might be disadvantaged.

The organization of political systems also has consequences for the strength of civil society, trust in the state, and *societal cohesion* (Pronkina et al., 2023; Shin, 2022). Open democracies, such as West Germany, foster participation and societal cohesion. East Germany, on the other hand, relied on citizen agents to spy on their friends, neighbors, and family members to detect opposition. Given existing evidence on the importance of social connectedness for cognitive health (Kuiper et al., 2016), this social pathway might imply a cognitive advantage for West German residents as compared to East German residents.

These economic and cultural arguments make it plausible to assume differences in cognitive functioning between older adults who were socialized or spent a majority of their working life in East vs. West Germany. Thus, in our first hypothesis, we assume that *individuals who were socialized in East Germany should exhibit worse cognitive functioning in later life (H1a)*.

### Education, Educational Systems, and Cognition

On the other hand, education plays a vital role in developing cognitive reserve (Lövdén et al., 2020; Schneeweis et al., 2014). Cognitive reserve is defined as the brain’s capacity to deal with age-related pathologies and slow cognitive decline (Singh-Manoux et al., 2012; Stern, 2002). Notably, evidence suggests stronger effects of education on *levels* of cognitive functioning in later life rather than on *slopes* or trajectories of age-related cognitive decline, yet there is reason to believe that differences between educational *systems* in particular may influence both of these to some extent (e.g., Lövdén et al., 2020). The division of East and West Germany, and its reunification more than 40 years later, also came with differences in schooling systems and the availability of early childhood education and care.

East Germany featured ten compulsory schooling years (6–16 years), while federal states in West Germany had 8 or 9 years of compulsory schooling (Hampf, 2019). Hence, East Germans experienced *a longer duration* of compulsory schooling. Compulsory schooling duration was harmonized and increased throughout West Germany, however. The East German schooling system was *less stratified* than the West-European one, reducing intergenerational transmission of education (Below et al., 2013). Besides a longer duration in compulsory schooling in East Germany, the use of *formal childcare* was widespread (Schober & Stahl, 2014), potentially further eliminating social class gaps in children’s cognitive skills (Blossfeld et al., 2011; Ghirardi et al., 2023; Kulic et al., 2019). Other studies even argue that the early enrollment in formal childcare may explain cognitive gaps in school children between East and West Germany (Skopek, 2017). Finally, Russian as a foreign language, and the implementation of a polytechnic secondary stage to foster excellence were parts of the curriculum in East German schools (Neuner, 1997), broadening the skills potentially learnt in East German schools. To sum up, longer exposure to early learning and schooling, and its higher demands both on the language and the mathematical side, as well as a less socially stratified schooling system might directly affect brain functioning and brain health in later life by offering more – and more varied – opportunities for brain development and cognitive stimulation.

Indeed, studies using time trends (Leist et al., 2021) and cohort variation (Rehnberg et al., 2024) in access to education, as well as studies treating educational reforms as quasi-natural experiments (Cappellari et al., 2023; Hampf, 2019; Karwowski & Milerski, 2021), show that more years of schooling are beneficial for cognitive functioning in mid-adulthood or later life. However, the cognitive benefits of more years of schooling seem non-linear, with disproportional impacts of reforms targeting the early and late school years.

Moreover, education and family policies may be interlinked, acting as life course policies (Scherger, 2024). The East German educational system was part of an investment-oriented welfare policy that aimed at fostering citizens’ human capital and work ability. It has been argued that – at similar levels of economic development – socialist states often provided more welfare services (particularly in the realms of education and healthcare), improving the “physical quality of life” (Cereseto & Waitzkin, 1989). Thus, despite a prevailing belief about negative consequences of maternal employment for children’s well-being in Germany (Sunström, 1999), recent research has shown that children who were born and socialized in East Germany report having experienced less childhood adversity (Schulz et al., 2022), and that attending formal childcare early on did not lead to higher mental health problems or somatic symptoms (Braunheim et al., 2024). The psychological advantage of attending ECEC is transmitted to the next generation, with children of East-Germany-born parents reporting having experienced less punitive parenting styles (Kasinger et al., 2025).

Together, these two arguments pertaining to brain-development and social mechanisms resonate with earlier studies on the impact of (early) education for cognition throughout the life course and in later life (Stern, 2002). Since the East German system is characterized by an early inclusion into the pedagogical system via widely-used formal childcare, and a longer retention in education via one additional year of compulsory schooling, this leads to our second hypothesis, which is counter to H1a. Specifically, we assume that *individuals who spent their educational career in East Germany should exhibit better cognitive functioning (H1b)*.

Not least, these two hypotheses are based on explanations from different fields and relate to different pathways into cognitive aging processes (physical and mental health on the one hand, suggesting higher vulnerability of East Germans; better access and longer exposure to schooling and learning opportunities on the other hand, advantaging East Germans). These two arguments suggest distinct mechanisms, which may operate simultaneously. Therefore, it is conceivable that both mechanisms may be at work, potentially offsetting each other. For that reason, our third hypothesis anticipates that *there is no difference in cognitive functioning between East and West Germans (H1c)*.

### A Life Course Perspective on Societal Impacts on Cognitive Functioning

These two explanations (better economic conditions, more civil liberties vs. longer and more egalitarian schooling) provide competing mechanisms, which likely operate simultaneously. Theoretically, both mechanisms could be in place, and offset each other. Moreover, depending on a person’s birth year, one or the other may work more strongly, due to its higher salience.

Based on life-course theory, we thus argue that *individual exposure to a system* might moderate the impact of political systems in two ways. We measure individual exposure to the system with age at re-unification, as it informs us about two things: first, the number of life years spent under the respective system, and secondly, in which system a person’s various life stages were spent. The life course perspective hosts two main mechanisms proposing that such information should be crucial: *duration *and *timing*. While the former posits that it matters *how long* the cumulative duration is spent under certain contextual circumstances, the other specifies *when* a contextual influence critically influences an outcome. The assumptions derived from these two propositions may be contradictory (for a summary, see e.g., Potente et al., 2021), and this also applies to our study.

First, following the idea of *cumulative (dis)advantage* (Dannefer, 2003; DiPrete & Eirich, 2006), small initial differences become larger over time, leading to growing gaps between advantaged and disadvantaged groups (i.e., between East and West residents, or vice versa), the longer they were exposed to their condition. This is evidenced by studies showing higher downward mobility (Below et al., 2013; Blossfeld, 2018) and fewer income rewards to education (Antoni & Heineck, 2012) for those born in East Germany. Based on the idea of cumulative exposure, we expect that *the older individuals were at the time of reunification, the larger the differences in cognitive functioning (H2a)*.

On the other hand, according to the idea of *critical periods* (Elder, 1994; Hallqvist et al., 2004), exposures affect outcomes more strongly in specific stages of the life course. Hunger and malnutrition have stronger impacts on cognitive abilities and health the earlier the exposure (Roseboom et al., 2011). Transitioning out of the less stratified East German educational system into an economically more rewarding, faster growing labor market might be beneficial in one’s twenties and early thirties (Maehler et al., 2024), while economic restructuring might matter less in the years directly preceding retirement. Conversely, mid-adulthood, with family formation and financial responsibilities, might limit the benefits of economic restructuring. In this phase, mental health seems particularly vulnerable (Berkman & Soh, 2017). Based on critical periods, we assume that *the older individuals were at the time of reunification, the smaller the differences in cognitive functioning between East and West residents (H2b)*.

## Research Design

### Data and Sample

Our research question requires data studying several cohorts, with varying durations of exposure to political systems. The Survey of Health, Ageing and Retirement in Europe (SHARE) is a longitudinal study of adults aged 50 or older across 28 European countries, including Germany. SHARE started collecting data in 2004, with refresher samples drawn in regular intervals. Respondents’ partners were surveyed, too. For the purpose of this study, we use the German subsample only. Our analysis of cognitive functioning draws on observations stemming from eight regular panel waves (waves 1 and 2, waves 4-9), that were collected between 2004 and 2021 in the German subsample. We examined both respondents and their partners if they were aged 50 or older. Each regular panel wave included cognitive testing, information on the household, employment situation, family relationships, and respondents’ physical and mental health. We retained all respondents who featured at least one regular panel interview (n = 21,909 person-year observations nested in n = 7,339 persons). Moreover, respondents were eligible for our analysis if they lived in either East or West Germany in 1989 (but not abroad), and if they were at least 50 years of age at the time of the interview (n = 21,327).

SHARE features a retrospective SHARELIFE life history interview, which was administered to the full sample in Wave 3 (2009) and to a partial sample of respondents who joined after Wave 3, in Wave 7 (2017). While our analysis of cognitive trajectories relies on regular panel interviews only, we used information from these retrospective SHARELIFE interviews to adjust for important confounding factors (see “control variables” below). Such information is available for n = 5,600 persons, which can be linked to n = 19,450 person-year observations. Our sample was further restricted by missing information on the control variables and the outcome variables. Our final analytical sample thus comprises n = 18,442 person-year observations (from n = 5,231 persons) meeting the eligibility criteria above and featuring valid information on the variables of interest. These respondents are observed over a period of up to 15 years, with an average of 4.16 years (SD: 4.01).

### Outcome Variables

We use four standardized cognitive tests to capture different aspects of cognitive functioning. *Working memory* is measured using the direct recall of a ten-word learning list (Ren et al., 2024). *Executive functioning* is measured using a semantic fluency test (“animal naming test”) (Ardila et al., 2006). Both tests are frequently applied to predict cognitive impairment and dementia (O’Donovan et al., 2023). *Orientation in time* is measured with correct answers on day, month, year, and day of the week, summed up in an index ranging from 1 (“very bad”) and 4 (“very good”). *Numeracy* tested respondents’ subtraction skills (repeated subtraction of “7” from “100” up to five times or until failure). Except for numeracy, all tests were measured in all waves; however, individual item non-response varied across survey waves and cognitive tests. As a consequence, case numbers vary between models with different outcome variables (see Table S2).

### Analytic Strategy

To detect “cognitive footprints” (Rossor & Knapp, 2015) of political systems, and disentangle mechanisms of cumulative disadvantage and critical periods, we study both *levels* and *changes* in cognitive functioning. Random effects growth curve models allow us to model individual trajectories over time, considering time-stable and time-varying predictors. All models include confounding and control variables (described below). Our analytic strategy encompasses two steps: First, we estimate East and West German trajectories by including an interaction term between the individual time-trend and a dichotomous indicator denoting where respondents lived in 1989 before reunification. In the second step, we test for the impact of age at reunification by including an interaction term between East/West residence in 1989 with age at reunification. Trends pertaining to time and age at reunification were estimated assuming both linear and non-linear associations. We present the models with better overall fit.

### Covariates

The covariates included in the models were carefully selected based on theory and previous research (e.g., Livingston et al., 2020). First, the relationship between educational attainment and cognitive aging is likely confounded by early-life factors (Ihle et al., 2016; Lövdén et al., 2020). We thus include retrospective accounts of self-rated health (up to age 10), cognitive ability (measured with self-rated mathematics and language skills as compared to peers) and the number of books in the parental household into our models. These variables were collected retrospectively in SHARELIFE interviews in waves 3 and 7. Models further control for respondent’s gender (1 = female), birth cohort (born before 1934, 1934–1945, 1946–1952, 1952–1959, born after 1959), highest educational level attained, grouped in three categories (“at most lower secondary education” representing ISCED 1 and 2, “upper secondary education (including higher education access certificates and vocational certificates)” representing ISCED 3 and 4, and “tertiary education” including vocational tertiary certificates, representing ISCED 5 and 6). We further adjust for the disposable household income measured in quintiles for each survey wave, residency in an urban or rural area (1 = urban), the presence of a partner in the household (1 = yes), and the total number of household members.

In the model estimating the role of age at reunification (the second research question), we replace our categorical variable comprising birth cohorts with a variable that measures the age at reunification (subtracting the birth year from the year 1989). This continuous variable ranging from 18 to 58 was then further grouped into nine categories to represent particular life stages which may represent critical periods: individuals who were in young adulthood (one category: up to age 29, representing the transition from school-to-work); mid-adulthood (four categories: 30–34, 35–39, 40–44, 45–59); later career (three categories: 50–54, 55–59, 60–65); and in retirement (one category: 66 or older).

## Results

**Figure 1.**
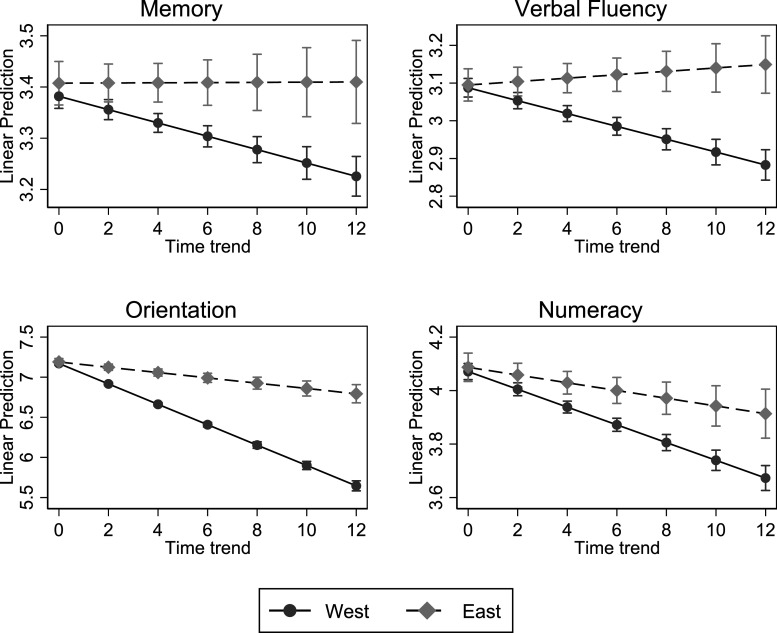
Time Trend in Decline by Living in East and West. *Note*. Random effects growth curve models, with interaction terms between East/West Germany and time trend. Confounders and control variables included. dependent variables are Z-standardized. Linear time trend. n (memory) = 18,232; n (verbal fluency) = 15,500; n (orientation) = 13,232; n (numeracy) = 13,735. For coefficients see Table S3 in the supplementary files)

Figure 1 shows predicted trajectories of cognitive functioning for respondents who lived in East and West Germany in 1989. Trajectories are displayed as individual time-trends, starting with the baseline observation (“0”). We find that respondents who lived in East Germany before the reunification exhibit better cognitive functioning overall. Compared to West Germans, they exhibit less steep declines in orientation and numerical abilities. Moreover, while West German residents exhibit decline over time in immediate memory and verbal fluency, East German residents remain stable or even slightly increase in these functions, leading to a growing gap in cognitive functioning. All interaction terms between time trend and residency in East Germany in 1989 are significant (*p* < 0.01; coefficients can be obtained in Table S2 in the supplementary files).

**Figure 2.**
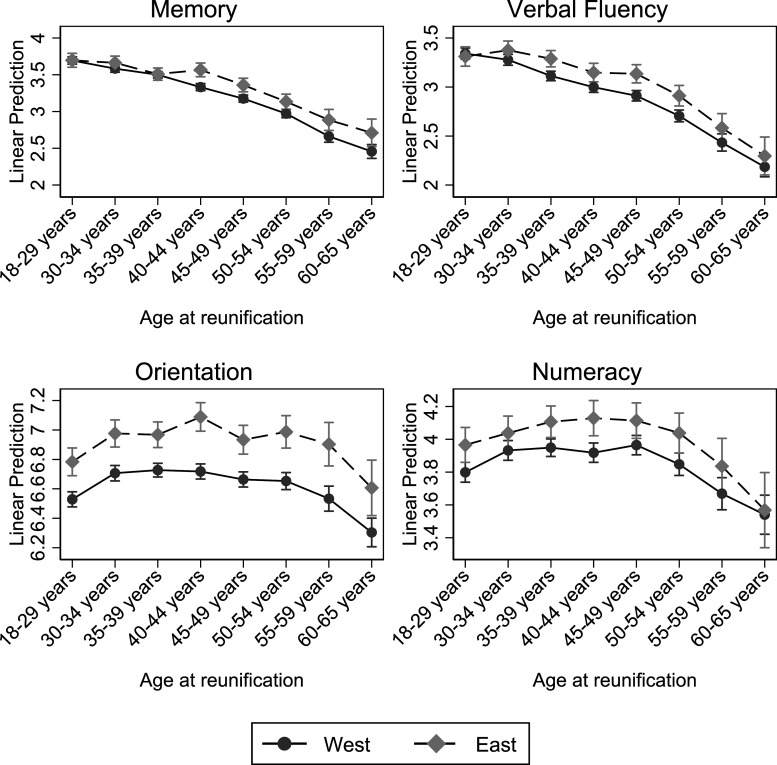
Predicted Cognitive Functioning by Age at Reunification. *Note*. Predicted probabilities from random effects growth curve models with interaction terms between residence in 1989 (East/West) and age at reunification. All respondents were at least 18 years old when the reunification took place. Dependent variables are Z-standardized. Models include control and confounding variables. Case numbers: (memory) = 18,232; n (verbal fluency) = 15,500; n (orientation) = 13,232; n (numeracy) = 13,735. For coefficients see Table S4

In the next step, we test our hypotheses regarding cumulative exposure to the political system and critical periods (Figure 2), measured with age at re-unification. For memory and verbal fluency, we find the largest gaps between persons who experienced reunification in their forties. This is also reflected in positive interaction terms between residency in East Germany in 1989 and age groups 40-44, 45-49 and 50-54 (see Table S4 in the supplementary files). Orientation and numeracy are higher among East Germans who experienced labor market entry during reunification (0.281*** and 0.185***), and East-West gaps persist across the different cohorts which experienced reunification at different ages. With the exception of orientation, cognitive skills converge among those who experienced reunification late in their careers. Overall these findings point to “critical periods”. While early labor market entry may have helped translating the educational benefit of East-German schooling into the West German labor market (setting East Germans apart in numeracy and orientation), family formation and economic competition in the wake of reunification in the forties may put a heavier burden on parents in West Germany (leading to greater gaps in memory and verbal fluency).

### Robustness Tests

To test for the robustness of our results, we estimated our models separately for men and women (see Figures S2 and S3 in the Supplementary Files). These analyses suggest that the differences observed between East and West Germans are larger among men, particularly for verbal fluency, orientation, and numeracy. Separate estimations pertaining to age at reunification are robust to the advantage of East-Germans in both genders.

We further examined the linearity of the association between the time trend and cognitive functioning (Tables S2 and S3). Results suggest that cognitive decline in West Germans follows an approximately linear trajectory beginning around five years after the first interview. Prior to that, particularly during the second and third interviews, cognitive scores are often higher, reflecting “learning effects” in cognitive tests such as memory or verbal fluency tests (Bartels et al., 2010). Beyond 12 years post-baseline, the linearity of cognitive trajectories becomes less distinct, though this may partly be due to small case numbers in certain time points, particularly for East Germans where case numbers are small.

Third, to examine differences by educational attainment, we included an interaction between education and residential location in 1989, the East German advantage remains stable over time for medium- and highly-educated individuals across all four outcome variables. Among the lower-educated, West Germans show an early advantage in memory, verbal fluency, and numeracy, though this gap narrows over time (Figure S5). Regarding critical periods, the stronger East-West gaps among those in their forties at reunification are concentrated among intermediate- and higher-educated individuals. For the lower-educated, age at reunification appears less influential (Figure S6).

Finally, given differences in physical health and mortality between East and West German residents, we estimated additional models controlling for physical health (measured the number of chronic conditions and the Global Activity Limitation Indicator (GALI), depression (using the EURO-D Index) and self-reported quality of life (using the CASP index)). Case numbers are reduced by 25–34%. The results regarding observed East-West differences observed (Table S5 in the supplementary files) are mostly unaffected by the inclusion of these variables. Similarly, we re-estimated our models excluding individuals who already have received a dementia diagnosis (Table S6). While case numbers are slightly reduced (3-7% depending on the outcome), the results remain stable.

Hence, selection into survey participation based on physical health or selective survival can be excluded as being the main drivers behind the observed differences. Yet, the positive interaction term between the time trend and being born or socialized in East Germany (denoting the advantage of exposure to the East German context) becomes insignificant for orientation when including these variables.

## Discussion

### Summary

The aim of this study was to examine the long-term implications of political system exposure on later life cognition, using the case study of German separation and later reunification. Based on differences in schooling systems and two key components of life course theory, we identified several mechanisms and examined trajectories of cognitive functioning, comparing individuals who lived in East- and West-Germany in 1989, and investigated whether age at reunification affected how pronounced East-West cognitive differences were.

Contrary to prior research concerning physical health in capitalist and post-socialist states (H1a), we did not find any evidence that West German individuals exhibited advantages in either levels or trajectories of cognitive health in later life. Supporting the competing hypothesis regarding the protective effect of the East German educational system (H1b), which included longer compulsory schooling and a less socially stratified educational system, we found overall higher cognitive benefits and slower decline among older Germans who had been socialized or spent a major part of their working lives in East Germany. This insight supports earlier findings on the importance of educational systems and length of schooling for building cognitive reserve (Leist et al., 2021; Schneeweis et al., 2014). Regarding the moderating potential of age at reunification, we find stronger support for the “critical periods” hypothesis (H2b) than for cumulative duration of exposure to the East-German system. These findings are in line with earlier studies on the impact of early opportunities and mid-life turbulences on later life health (Berkman & Soh, 2017; Roseboom et al., 2011). After conducting various robustness tests eliminating potential bias through selective mortality, we are confident the benefits experienced by East Germans can be attributed to the contextual conditions.

Robustness tests confirm that these associations are stable for men and women, and independent of selective participation based on physical and mental health. Overall, our findings pertaining to time trends and duration of exposure were similar across measures (memory, executive functioning, orientation, and numeracy). The only exception is orientation in time, where we found no sign of convergence among adults who experienced reunification in their late careers, but a persisting advantage of respondents who lived in East Germany in 1989.

### Limitations and Outlook for Future Research

Despite the notable contributions that this study makes, there are a number of limitations. First, while we make a theoretically based and empirically grounded argument that differences between East and West Germans are likely due to differences in access to and quality of education, other differences between East and West Germany existed as well. Among others, exposure to environmental toxins such as lead in gasoline (which was higher in West Germany due to the rarity of cars in East Germany), and the use of drugs for recreational use (also higher in West Germany than in East Germany), but also on the other hand trust in civil society and protection from institutional arbitrariness (higher in West Germany than in East Germany) might also affect brain health and cognitive functioning in later life. Moreover, psychosocial determinants of cognitive health, including control beliefs and social cohesion, may vary across political systems, as well, with potential implications for later life trajectories of cognitive functioning (e.g., Diewald, 2007). Since not all of these factors are measurable at an individual level (e.g., societal cohesion), nor were others available in the data used (e.g., control beliefs), our analysis does not allow us to disentangle the various explanatory mechanisms or isolate the respective contribution(s) of such psychosocial factors.

Second, we have focused on the comparison of East and West Germany, exploiting their cultural similarity before the division, and their shared legal regulations and labor markets after reunification. Yet, to test for the robustness of our claims and arguments, it would be desirable to replicate our study in a comparison of East and West European studies. The SHARE Europe-wide data set would allow researchers to implement such a research design; however, the experimental component would be weakened, as other post-socialist states sampled in SHARE do not have a clear counterpart. Future research could expand our research question, examining differences in cognitive functioning both between and within post-socialist countries, and explain differences in cognition via differences in the countries’ respective educational systems, labor markets and policies after the transition to market economy.

Third, this study posits opposite hypotheses for both the differences between East and West Germany, and the role of respondents’ age at reunification. Arguments are derived from plausible theoretical assumptions. How and why one mechanism is predominant over the other remains unclear and needs more research^1^. Moreover, prior research has noted that ceiling and floor effects of cognitive tests (Franco-Marina et al., 2010; Scollard et al., 2023) may play a role in determining results of studies such as ours; for example, those with higher starting levels of cognitive functioning may have steeper slopes of decline in later life, in part because they have *greater cognitive reserve* (Aartsen et al., 2019), or higher tolerance of pathologies until reaching the threshold to dementia (Scarmeas, 2005), before becoming unable to complete survey data collection and cognitive testing. Future research that incorporates onset of Alzheimer’s disease and other dementia related diseases (ADRD) as alternative outcomes, in addition to attrition due to physical health problems and mortality, would address these concerns and are hereby encouraged.

## Conclusions

In line with earlier studies on cognitive aging and dementia (Leist et al., 2021; Schneeweis et al., 2014), results of this study underscore the importance of (early life) education for later life cognitive health. As our study shows, the advantage of educational exposure for later life cognitive functioning holds also in the context of a political system in East Germany. These results are surprising in two respects, and represent a paradox. First, they depart from the established pattern that educational attainment influences levels of later-life cognitive function but not slopes or speed of decline (Lövdén et al., 2020). This may be explained by the fact that our quasi-experimental design captures the potential imprints of the educational system, not individual educational degrees, or participation in higher education (which were more prevalent in East Germany but are adjusted for in our models).

Secondly, it establishes a paradox, as having grown up or lived in East Germany has been linked with poorer health behaviors, physical health, and earlier mortality (Grigoriev & Pechholdová, 2017; Heinemann et al., 1996). However, similar patterns of lower physical but better cognitive health among older adults were also found in studies comparing East, South, and West European countries (Abuladze et al., 2023), mollifying the “Eastern disaster” (Mackenbach, 2017). Nevertheless, given the novelty of our findings, the broader research on political systems and their impact on cognitive functioning warrants more research.

To date, research on the long-term health consequences of different socialization and exposure in East and West Germany, and their mechanisms, remains sparse. Notable exceptions have addressed mental health and well-being (Bister et al., 2023; Kasinger et al., 2023), and physical health (Bister et al., 2022; Grigoriev & Pechholdová, 2017). Similarly, research on institutional conditions and life course outcomes in post-socialist countries is often sparse, due to a lack of suitable longitudinal data (representing both general populations and older adults). Recent advancements in the funding landscape (e.g., mandatory inclusion of post-socialist countries in EU-funded COST Actions), and the inclusion of many post-socialist countries in the European SHARE data, are promising avenues to remedy this research gap.

Despite the need for further research, some first preliminary conclusions can be drawn. Given the fast-growing life expectancy and population aging in many lower- and middle-income countries (Salomon et al., 2012; Wang et al., 2020), among them many with less comprehensive or more unequal (early) education and schooling systems (Local Burden of Disease Educational Attainment Collaborators, 2020; Lu et al., 2020; Reinders et al., 2021), there is a need to understand which critical factors foster cognitive abilities and preserve cognitive functioning into the years of later life. This is important in the context of rising authoritarianism, which is often openly hostile to educational systems (Azevedo & Robertson, 2022; Dönmez & Duman, 2021; Doğan & Selenica, 2022; Giudici, 2021). As numerous countries worldwide undergo substantial changes to their respective political systems, this is likely to have disparate impacts upon different age groups and cohorts within each social and political setting, with implications for cognitive health and healthcare systems in the future.

Our study highlights the importance of taking a life course approach to studying social determinants of health, as age at reunification played a critical role in determining the long-term benefits of experiencing the East German educational system. This points to the idea of critical periods (Hallqvist et al., 2004; Kwon et al., 2018). Finally, results of our study underscore the nuanced advantages and disadvantages of different political systems for population health. They highlight that advantageous settings for accumulating cognitive reserve throughout childhood, adolescence, early and mid-adulthood, are those with both strong and egalitarian public educational systems *and* opportunities for economic advancement and job security. Comparing a planned with a liberal market economy, we conclude that neither East nor West Germany provided its population with both.

## Supplemental Material

Supplemental Material - Is There a Cognitive Footprint of Political Systems? The Case of Separation and Reunification of East and West Germany and Its Association With Later Life Cognitive Health Supplemental Material for Is There a Cognitive Footprint of Political Systems? The Case of Separation and Reunification of East and West Germany and Its Association With Later Life Cognitive Health by Ariane Bertogg, Jeffrey E. Stokes in Research on Aging

## Data Availability

This paper uses data from SHARE Waves 1, 2, 3, 4, 5, 6, 7, 8 and 9 (DOIs: 10.6103/SHARE.w1.900, 10.6103/SHARE.w2.900, 10.6103/SHARE.w3.900, 10.6103/SHARE.w4.900, 10.6103/SHARE.w5.900, 10.6103/SHARE.w6.900, 10.6103/SHARE.w6. DBS.100, 10.6103/SHARE.w7.900, 10.6103/SHARE.w8.900, 10.6103/SHARE.w8ca.900, 10.6103/SHARE.w9.900, 10.6103/SHARE.w9ca900, 10.6103/SHARE.HCAP1.100) see Börsch-Supan et al. (2013) for methodological details. The SHARE data collection has been funded by the European Commission, DG RTD through FP5 (QLK6-CT-2001-00360), FP6 (SHARE-I3: RII-CT-2006-062193, COMPARE: CIT5-CT-2005-028857, SHARELIFE: CIT4-CT-2006-028812), FP7 (SHARE-PREP: GA N211909, SHARE-LEAP: GA N227822, SHARE M4: GA N261982, DASISH: GA N283646) and Horizon 2020 (SHARE-DEV3: GA N676536, SHARE-COHESION: GA N870628, SERISS: GA N654221, SSHOC: GA N823782, SHARE-COVID19: GA N101015924) and by DG Employment, Social Affairs & Inclusion through VS 2015/0195, VS 2016/0135, VS 2018/0285, VS 2019/0332, VS 2020/0313, SHARE-EUCOV: GA N101052589 and EUCOVII: GA N101102412. Additional funding from the German Federal Ministry of Education and Research (01UW1301, 01UW1801, 01UW2202), the Max Planck Society for the Advancement of Science, the U.S. National Institute on Aging (U01_AG09740-13S2, P01_AG005842, P01_AG08291, P30_AG12815, R21_AG025169, Y1-AG-4553-01, IAG_BSR06-11, OGHA_04-064, BSR12-04, R01_AG052527-02, R01_AG056329-02, R01_AG063944, HHSN271201300071C, RAG052527A) and from various national funding sources is gratefully acknowledged (see https://www.share-eric.eu/).

## Note

1 To encourage reproduction of our analyses, we will provide reproducibility code after acceptance.
