# Supplementary material for: Is There a Cognitive Footprint of Political Systems? The Case of Separation and Reunification of East and West Germany and Its Association With Later Life Cognitive Health
Source: Res Aging. 2025 Oct 4;48(7-8):351–64. doi: 10.1177/01640275251383810 (PMC13260745; doi:10.1177/01640275251383810)
Supplement: Supplemental Material - Is There a Cognitive Footprint of Political Systems? The Case of Separation and Reunification of East and West Germany and Its Association With Later Life Cognitive Health [file sj-pdf-1-roa-10.1177_01640275251383810.pdf]

Is there a cognitive footprint of political systems? The case of separation and reunification of East and West Germany and its association with later life cognitive health.

Supplementary Materials

Figures

Figure S.1: Residence in 1989 and when surveyed

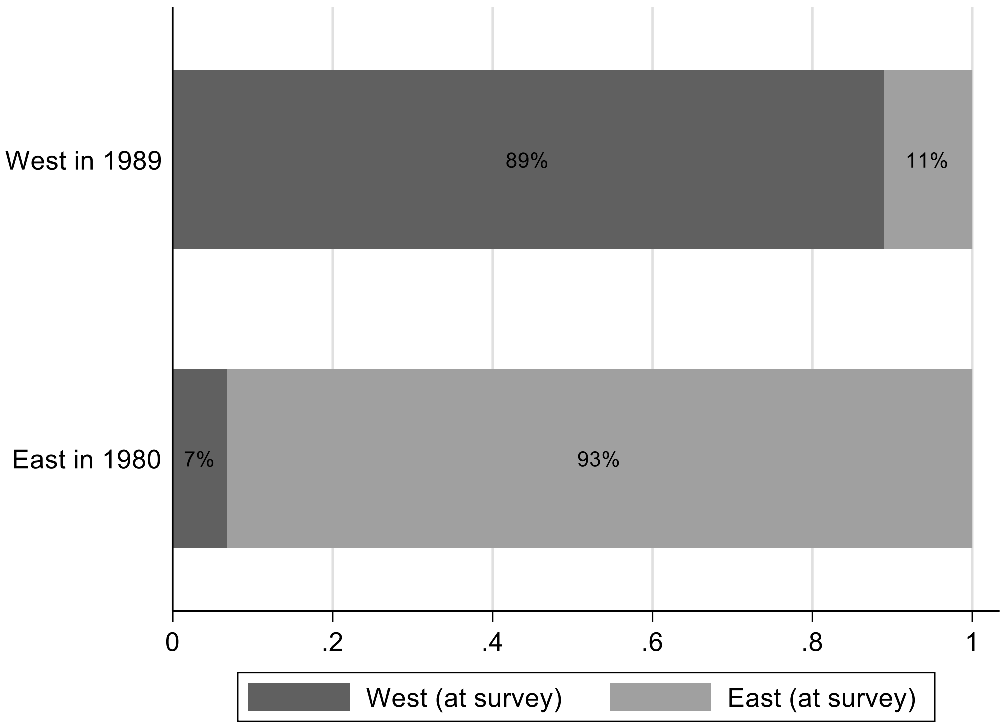

Figure S.2: Time Trend by Gender (linear specification)

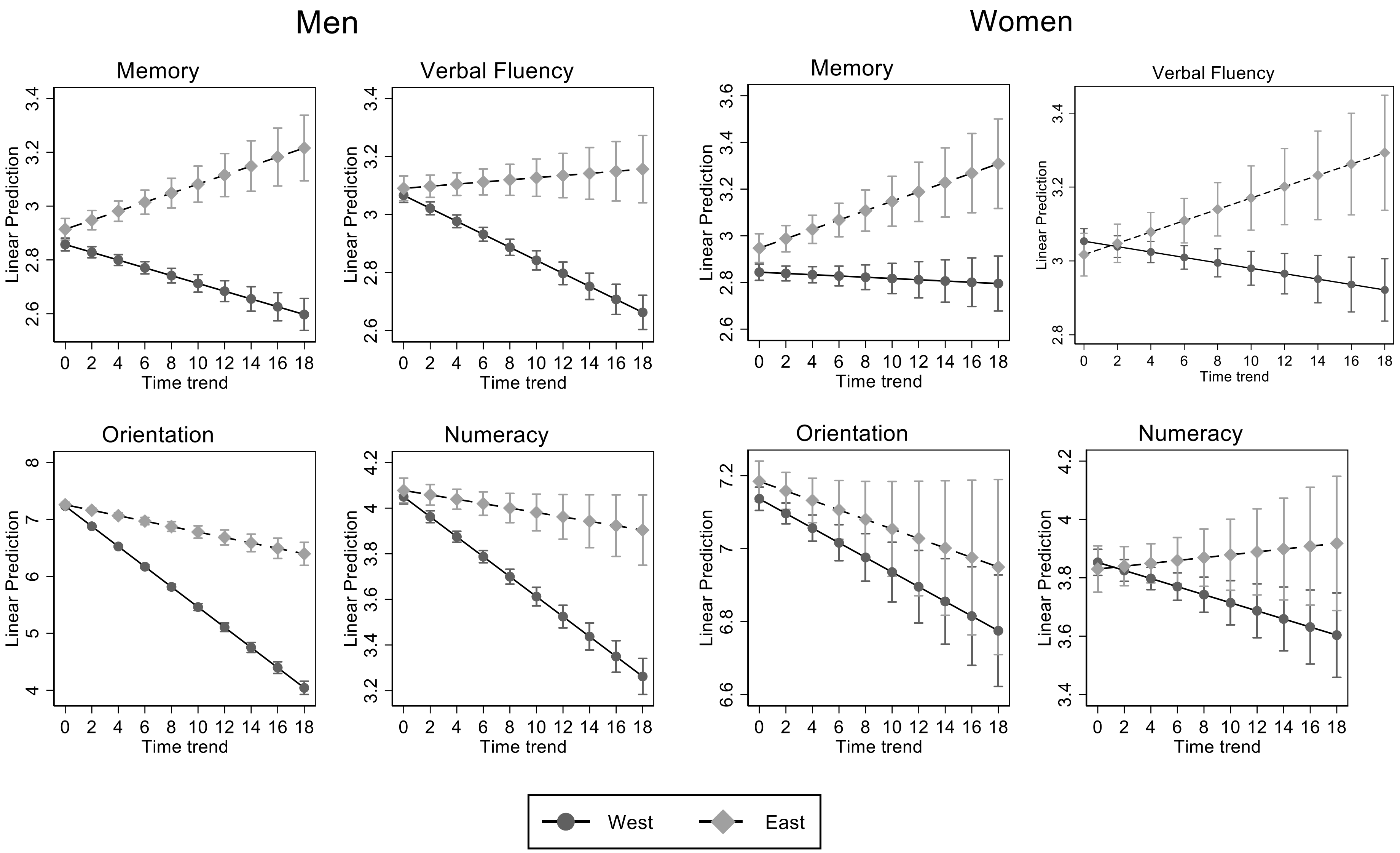

Figure S.3: Age at Reunification by Gender (non-linear specification)

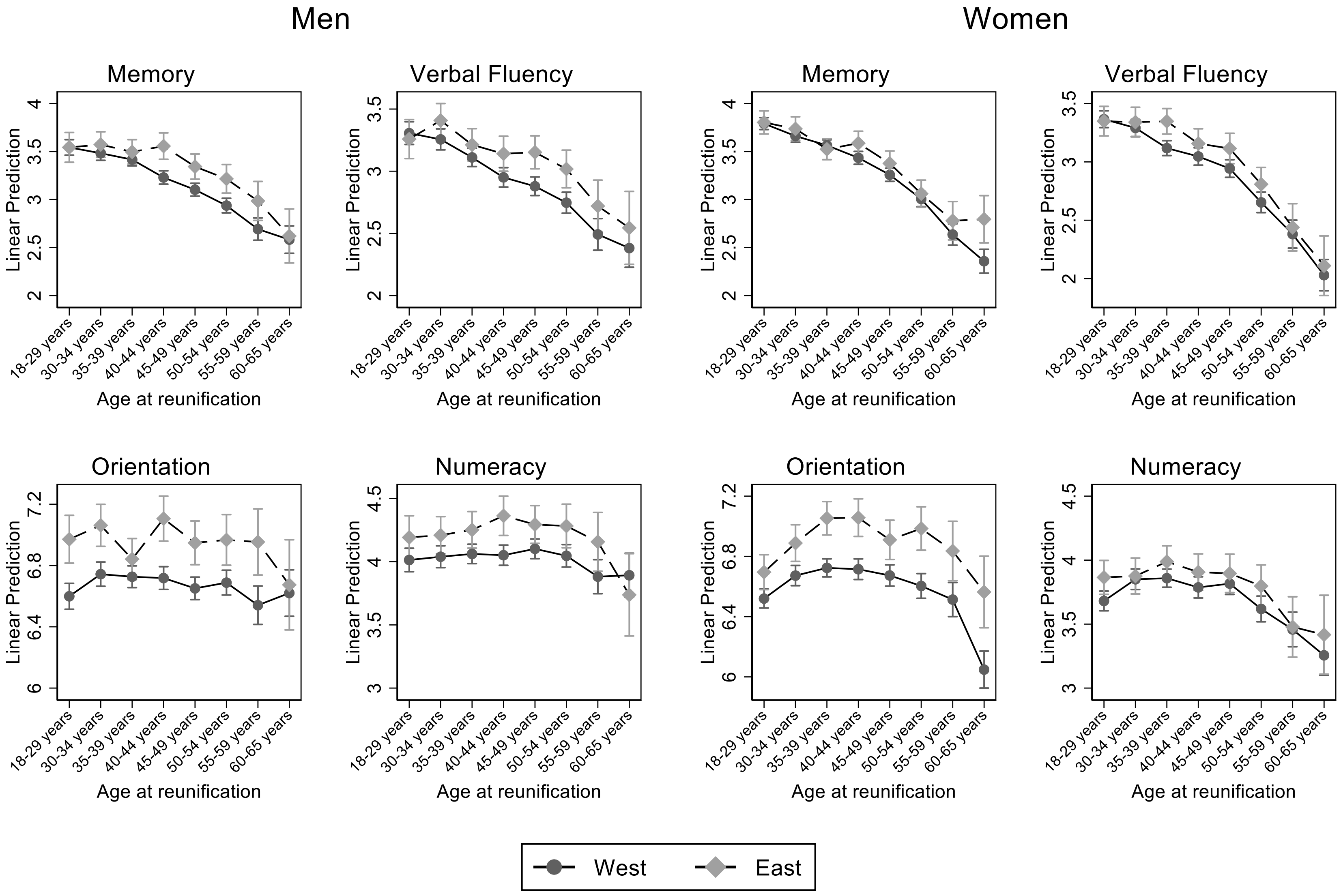

Figure S.4: Density Plots – Distribution of the Dependent Variables

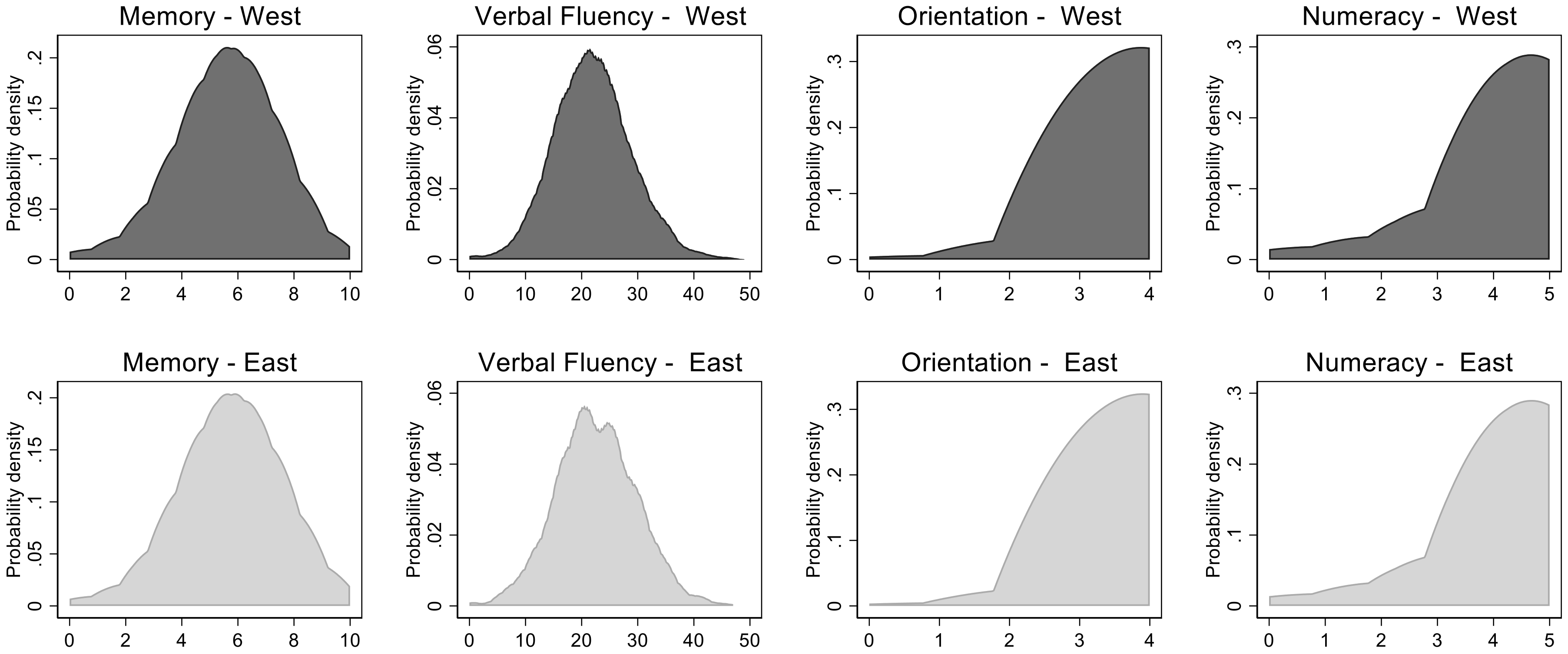

Figure S.5: Time Trend by Educational Level

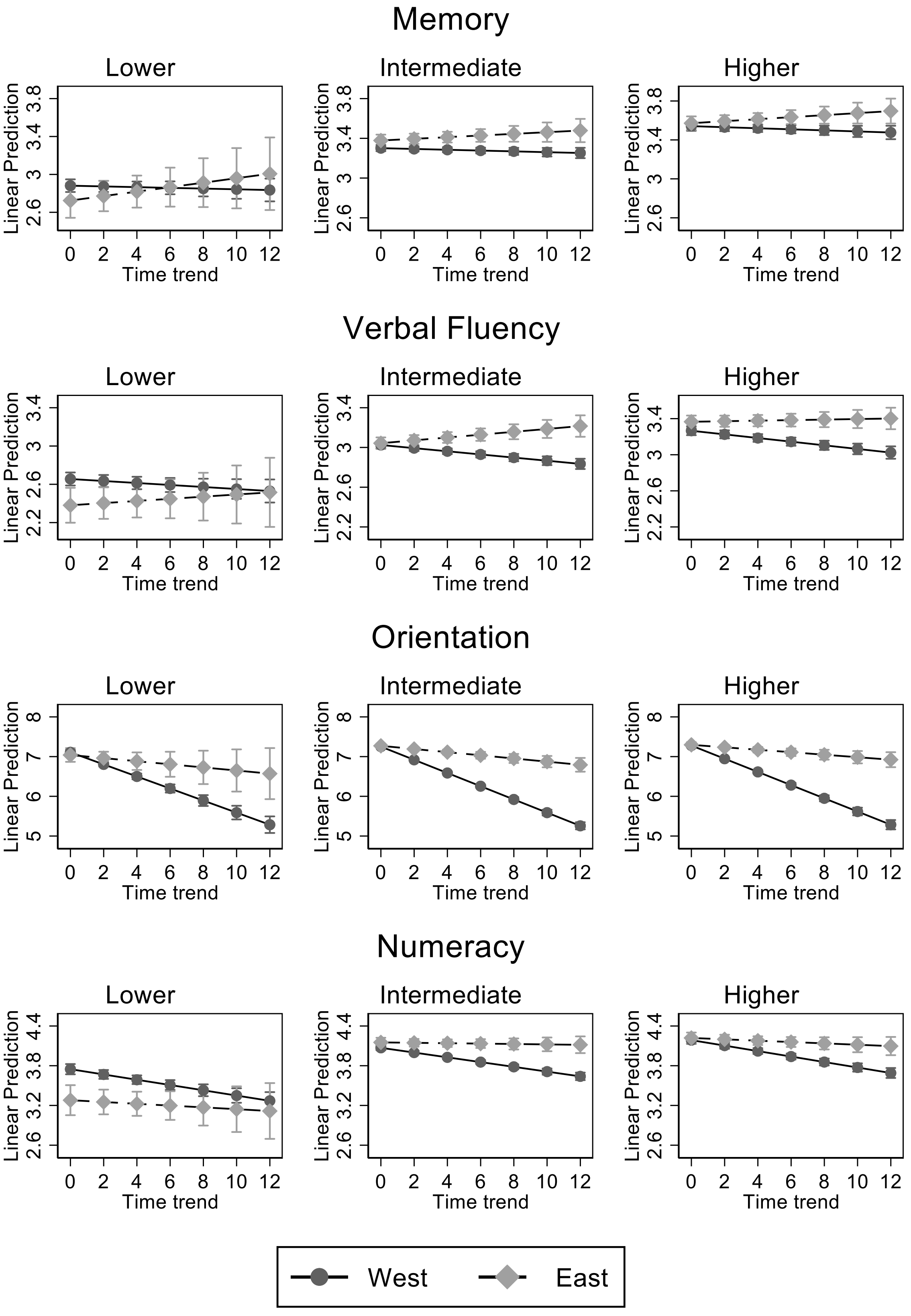

Figure S.6: Age at Reunification by Educational Level

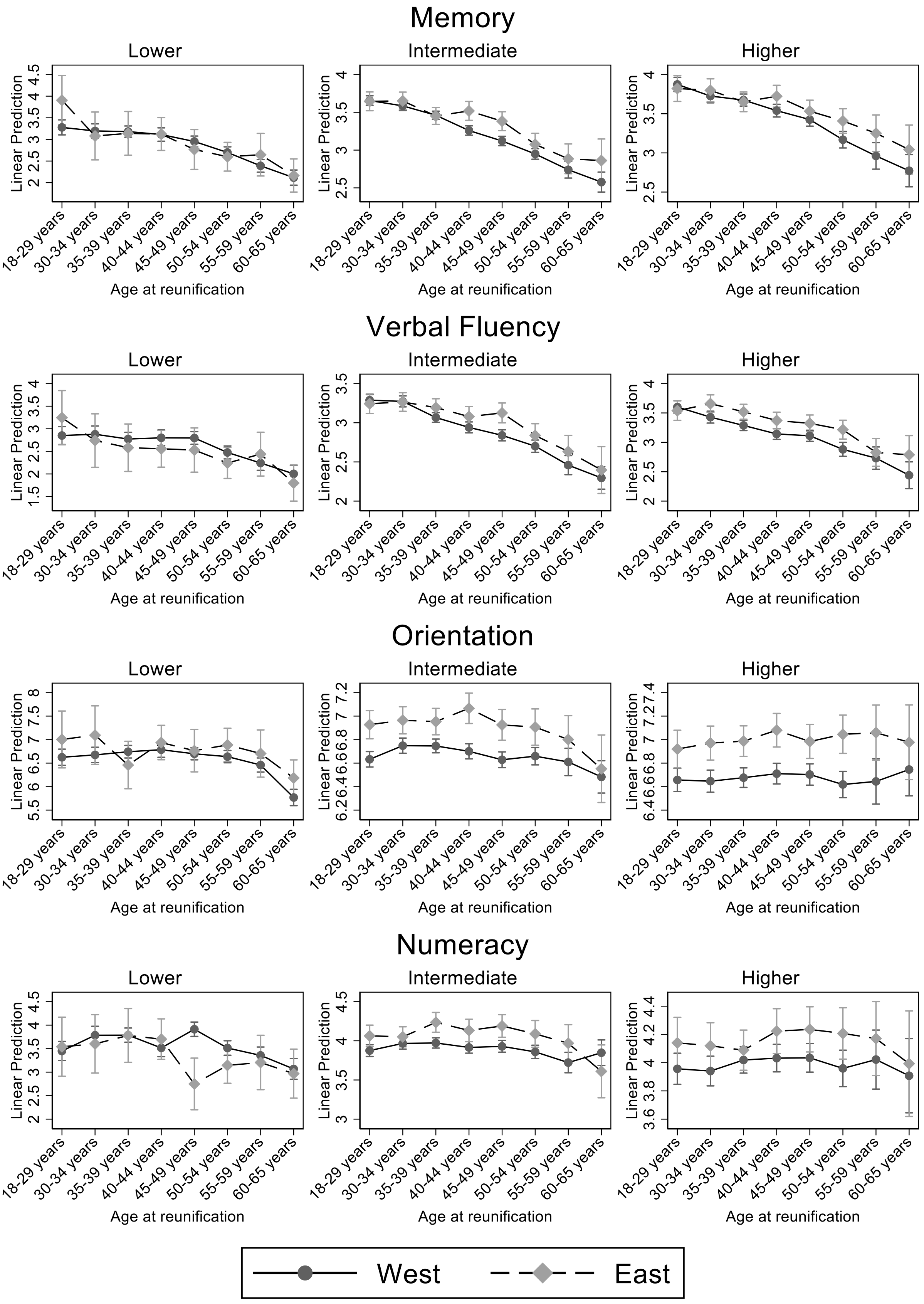

Tables

Table S.1: Sample Description

Sample	**All**		**West**		**East**	
Variable	Mean	SD	Mean	SD	Mean	SD
Dependent Variables – Original Scale						
Orientation	3.836	.533	3.83	.543	3.862	.487
Numeracy	4.426	1.11	4.421	1.115	4.446	1.091
Memory	5.611	1.696	5.587	1.687	5.723	1.733
Verbal Fluency	22.117	7.294	21.996	7.232	22.604	7.521
Standardized Scale						
Orientation	6.819	1.13	6.747	1.161	7.128	.922
Numeracy	3.94	1.012	3.921	1.014	4.018	.999
Memory	3.32	1.001	3.308	.997	3.376	1.018
Verbal Fluency	3	.99	2.979	.979	3.084	1.027
East vs. West						
East at interview	.245	.43	.105	.306	.928	.258
East	.17	.376	0	0	1	0
West	.83	.376	1	0	0	0
Other treatment variables						
Age at reunification	42.921	9.461	42.841	9.318	43.311	10.123
Confounders: Childhood						
Number of books in parental home (age 10)	-6.894	7.509	-6.759	7.541	-7.552	7.311
Math skills (relative to peers, age 10)	-6.722	7.654	-6.584	7.687	-7.398	7.453
Language skills (relative to peers, age 10)	-6.796	7.61	-6.665	7.642	-7.436	7.416
Childhood health (self-reported, up to age 15)						
Excellent	.197	.397	.2	.4	.179	.384
Very good	.315	.465	.314	.464	.322	.467
Good	.354	.478	.348	.476	.382	.486
Fair	.103	.304	.106	.307	.092	.289
Poor	.026	.158	.027	.162	.02	.141
Health varied a lot	.005	.072	.005	.073	.004	.065
Age at interview	66.239	9.843	66.41	9.836	65.405	9.836
Control Variables						
Birth Cohort						
born before 1934	.067	.251	.067	.249	.07	.256
born between 1934 and 1945	.199	.399	.197	.398	.208	.406
born between 1946 and 1952	.319	.466	.319	.466	.316	.465
born between 1953 and 1959	.311	.463	.31	.462	.314	.464
born after 1959	.104	.306	.108	.31	.092	.289
Gender: woman	.523	.499	.518	.5	.539	.499
Educational Level						
At most lower secondary	.132	.339	.151	.358	.071	.257
Upper secondary (academic,	.57	.495	.579	.494	.541	.498

vocational)						
Tertiary	.297	.457	.27	.444	.388	.487
Lives in urban area	.335	.472	.337	.473	.33	.47
Partner in household	.758	.428	.759	.428	.755	.43
Household size	2.013	.788	2.044	.825	1.911	.643
Self-perceived health:						
Excellent	.053	.224	.061	.24	.027	.161
Very good	.145	.352	.158	.365	.101	.302
Good	.404	.491	.4	.49	.417	.493
Fair	.303	.46	.292	.455	.342	.474
Poor	.095	.293	.089	.285	.114	.317
Household income (disposable, annual)						
Lowest quintile	.273	.445	.285	.452	.212	.409
Second quintile	.246	.431	.222	.416	.363	.481
Third quintile	.24	.427	.237	.425	.257	.437
Highest quintile	.24	.427	.255	.436	.168	.374
N			17352		3601	

Note: Mean and standard deviations are reported for continuous variables. Shares (percentages) are reported for categorical variables

Table S.2: Coefficients – Linear Time Trend

	(1) Memory	(2) Verbal Fluency	(3) Orientation	(4) Numeracy
East	0.026	0.007	0.020	0.016
Time trend	-0.013***	-0.017***	-0.127***	-0.033***
East * Time trend	0.013**	0.022***	0.094***	0.019***
Age at baseline	-0.035***	-0.032***	-0.032***	-0.020***
Number of books (age 10)	0.069***	0.086***	0.019*	0.038***
Math skills (relative to others, age 10)	-0.084***	-0.068***	-0.036**	-0.149***
Language skills (relative, age 10)	-0.075***	-0.073***	-0.011	-0.024
Childhood health: Excellent				
Very good	-0.003	-0.020	-0.022	0.026
Good	-0.054*	-0.070*	-0.022	-0.003
Fair	-0.072*	-0.112**	-0.014	-0.010
Poor	-0.089	-0.038	-0.044	-0.038
Varied a lot	0.069	-0.101	-0.033	-0.041
Birth cohort: born before 1934				
born between 1934 and 1945	0.134***	0.140***	0.017	0.025
born between 1946 and 1952	0.083*	-0.020	-0.704***	-0.300***
born between 1953 and 1959	0.047	0.080	-0.307***	-0.173**
born after 1959	0.005	0.003	-0.469***	-0.275***
Female	0.173***	0.058**	-0.045*	-0.266***
Educational level: At most lower secondary				
Upper secondary (academic, vocational)	0.303***	0.268***	0.087**	0.362***
Tertiary	0.540***	0.526***	0.137***	0.460***
Lives in urban area	0.021	-0.050**	0.009	0.024
Lives with partner or spouse	0.014	0.078**	0.139***	0.009
Size of household	-0.022*	-0.045***	-0.066***	-0.042**
Income: Lowest quintile				
Second quintile	-0.012	0.098***	0.043	0.180***
Third quintile	0.044*	0.150***	0.008	0.202***
Highest quintile	0.052**	0.184***	-0.008	0.206***
Lives in urban area	0.080***	0.063**	0.004	0.100***
Constant	5.268***	4.738***	9.556***	5.457***
lns1_1_1				
Constant	-3.631***	-3.408***	-2.444***	-3.547***
lns1_1_2				
Constant	-0.715***	-0.486***	-1.069***	-0.550***
lnsig_e				
Constant	-0.340***	-0.527***	-0.265***	-0.344***
Observations	18232	15500	13232	13735

Table S.3: Coefficients – Non-Linear Time-Trend (Categorical Variable)

	(1)<br>Memory	(2)<br>Verbal Fluency	(3)<br>Orientation	(4)<br>Numeracy
East	0.072**	-0.001	0.035	0.034
Baseline survey (year 0; ref)				
2	0.043*	0.040*	0.011	0.047*
3	-0.001	0.057	-0.055	
4	0.159***	-0.204***	-0.843***	-0.110*
5	0.119	0.146		-0.281
6	-0.008	-0.171***	-1.691***	-0.390***
7	-0.101**	-0.188***	-0.685	-0.039
8	-0.115*	-0.166***	-0.131*	-0.317***
9	-0.149***	-0.131***	0.021	-0.111*
10	-0.040	-0.096	-0.304***	-0.264***
11	-0.181***	-0.099**	-0.126**	-0.115*
12	-0.204***	-0.380***	-1.812***	-0.625***
13	-0.215***	-0.125**	-0.264***	-0.295***
15	-0.230***	-0.279***	-1.789***	-0.461***
East * 2	0.011	0.056	-0.056	-0.050
East * 3	-0.056	-0.168*	-0.005	
East * 4	-0.325***	0.093	-0.631	-0.093
East * 5	-0.138	0.057		-1.138*
East * 6	0.074	0.192*	0.311	0.245*
East * 7	0.063	0.057	0.000	-0.122
East * 8	0.088	0.272**	-0.024	0.132
East * 9	0.100	0.147*	0.000	0.087
East * 10	0.085	0.142	0.111	-0.026
East * 11	0.235**	0.136*	0.042	0.033
East * 12	n.a.	n.a.	n.a.	n.a.
East * 13	0.110	0.209**	0.061	0.204*
East * 15	-0.460	1.012	-2.232*	0.184
Age at baseline	-0.036***	-0.032***	-0.020***	-0.017***
Number of books (age 10)	0.067***	0.087***	0.018*	0.040***
Math skills (relative to others, age 10)	-0.086***	-0.069***	-0.033**	-0.149***
Language skills (relative, age 10)	-0.070***	-0.069***	-0.004	-0.020
Childhood health: Excellent				
Very good	-0.003	-0.018	-0.013	0.025
Good	-0.055*	-0.067*	-0.013	-0.002
Fair	-0.070*	-0.113**	-0.005	-0.013
Poor	-0.082	-0.034	-0.038	-0.048
Varied a lot	0.083	-0.099	-0.005	-0.056
Birth cohort: born before 1934				
born between 1934 and 1945	0.116**	0.123**	0.091*	0.044
born between 1946 and 1952	0.051	0.022	-0.011	-0.117*
born between 1953 and 1959	0.005	0.047	-0.067	-0.114*
born after 1959	-0.044	-0.042	-0.158**	-0.217**
Female	0.173***	0.060**	-0.036*	-0.264***
Educational level: At most lower secondary				

Upper secondary (academic, vocational)	0.287***	0.268***	0.096***	0.366***
Tertiary	0.516***	0.524***	0.148***	0.461***
Lives in urban area	0.021	-0.048*	-0.000	0.025
Lives with partner or spouse	-0.022	0.073**	0.068**	-0.015
Size of household	-0.025*	-0.043**	-0.059***	-0.035*
Income: Lowest quintile				
Second quintile	0.073***	0.098***	0.073**	0.182***
Third quintile	0.145***	0.152***	0.068*	0.211***
Highest quintile	0.162***	0.189***	0.070*	0.221***
Lives in urban area	0.076***	0.062**	-0.018	0.092***
Constant	5.283***	4.756***	8.475***	5.168***
lns1_1_1				
Constant	-3.640***	-3.416***	-3.051***	-3.612***
lns1_1_2				
Constant	-0.724***	-0.487***	-1.009***	-0.553***
lnsig_e				
Constant	-0.341***	-0.532***	-0.362***	-0.351***
Observations	18232	15500	13232	13735

Table S.4: Coefficients – By Age at Reunification

	(1) Memory	(2) Verbal Fluency	(3) Orientation	(4) Numeracy
East	-0.005	-0.032	0.281***	0.185**
Age at reunification: 18-29 years (ref):				
30-34 years	-0.097**	-0.062	0.072*	0.079
35-39 years	-0.189***	-0.240***	0.085*	0.110**
40-44 years	-0.358***	-0.358***	0.068	0.050
45-49 years	-0.486***	-0.418***	0.021	0.106*
50-54 years	-0.695***	-0.608***	0.008	-0.011
55-59 years	-0.926***	-0.820***	-0.056	-0.112
60-65 years	-1.126***	-1.031***	-0.318***	-0.149*
Retired	-1.402***	-1.307***	-1.001***	-0.522***
East * 30-34 years	0.069	0.115	-0.030	-0.087
East * 35-39 years	-0.012	0.200**	-0.056	-0.028
East * 40-44 years	0.220**	0.186**	0.071	0.022
East * 45-49 years	0.182*	0.257***	-0.006	-0.020
East * 50-54 years	0.158*	0.217**	0.032	-0.002
East * 55-59 years	0.224*	0.177	0.021	0.026
East * 60-65 years	0.235*	0.137	0.012	-0.245
East * Retired	-0.245	-0.255	-0.175	-1.320***
Time trend	-0.002	-0.013***	-0.149***	-0.034***
Number of books (age 10)	0.071***	0.091***	0.033***	0.048***
Math skills (relative to others, age 10)	-0.089***	-0.073***	-0.044***	-0.154***
Language skills (relative age 10)	-0.076***	-0.074***	-0.007	-0.019
Childhood health: Excellent				
Very good	-0.001	-0.021	-0.028	0.020
Good	-0.056*	-0.077**	-0.040	-0.014
Fair	-0.074*	-0.121**	-0.030	-0.018
Poor	-0.097	-0.053	-0.102	-0.066
Varied a lot	0.093	-0.064	0.008	-0.031
Gender: Woman	0.181***	0.064**	-0.034	-0.251***
Education: low				
Education: intermediate	0.292***	0.252***	0.072*	0.354***
Education: high	0.520***	0.499***	0.083*	0.432***
Lives in urban area	0.030	-0.040*	0.018	0.029
Lives with partner or spouse	0.001	0.062*	0.032	-0.030
Size of household	-0.013	-0.029*	0.003	-0.001
Income: Lowest quintile				
Second quintile	0.001	0.091***	0.017	0.166***
Third quintile	0.064***	0.142***	-0.004	0.190***
Highest quintile	0.083***	0.180***	0.011	0.207***
Lives in urban area	0.080***	0.070***	0.015	0.104***
Constant	3.491***	3.139***	7.165***	3.939***
lns1_1_1 Constant	-3.653***	-3.346***	-2.143***	-3.469***
lns1_1_2 Constant	-0.717***	-0.491***	-1.822***	-0.552***
lnsig_e Constant	-0.337***	-0.523***	-0.193***	-0.335***
Observations	18232	15500	13232	13735

Table S.5: Robustness Analysis – Controlling for Physical and Mental Health

	(1) Orientation	(2) Numeracy	(3) Memory	(6) Verbal Fluency
East	0.015	-0.045	0.081**	0.031
Time trend	-0.013***	-0.014***	-0.012***	-0.011***
East * Time trend	0.002	0.012*	0.013**	0.023***
Age at baseline	-0.019***	-0.017***	-0.026***	-0.021***
Number of books (age 10)	0.009	0.023*	0.056***	0.082***
Math skills (relative to others, age 10)	-0.022*	-0.143***	-0.067***	-0.061***
Language skills (relative, age 10)	-0.009	-0.027*	-0.070***	-0.080***
Childhood health (up to age 15): excellent				
Very good	-0.006	0.037	0.032	-0.010
Good	0.019	0.028	0.005	-0.055
Fair	0.047	0.040	-0.005	-0.072
Poor	0.007	0.021	-0.006	0.042
Varied a lot	0.135	-0.079	0.083	-0.095
Birth cohort: born before 1934				
born between 1934 and 1945	0.025	0.023	0.175***	0.230***
born between 1946 and 1952	-0.060	-0.100	0.209***	0.241***
born between 1953 and 1959	-0.116	-0.122	0.237**	0.330***
born after 1959	-0.205*	-0.230*	0.225*	0.308***
Female	-0.046*	-0.291***	0.148***	0.060**
Education: low	0.000	0.000	0.000	0.000
Education: intermediate	0.028	0.321***	0.221***	0.220***
Education: high	0.069*	0.403***	0.432***	0.454***
Lives in urban area	-0.017	0.017	0.020	-0.039
Lives with partner or spouse	0.076**	0.000	0.064*	0.045
Size of household	-0.036**	-0.024	-0.041**	-0.023
Income: Lowest quintile (ref):				
Second quintile	0.007	0.133***	0.075**	0.074**
Third quintile	-0.005	0.151***	0.159***	0.133***
Highest quintile	-0.013	0.143***	0.168***	0.181***
Lives in urban area	-0.048*	0.059*	0.024	0.030
Limitations with activities – GALI	-0.028	-0.029	-0.026	-0.008
Number of chronic conditions	-0.010	0.004	-0.001	-0.008
Depression (EURO-D)	-0.023***	-0.031***	-0.037***	-0.020***
Quality of Life (CASP)	0.006**	0.010***	0.010***	0.015***
Constant	8.386***	5.014***	4.301***	3.457***
lns1_1_1				
Constant	-3.616***	-4.068***	-4.308***	-3.383***
lns1_1_2				
Constant	-1.205***	-0.598***	-0.785***	-0.491***
lnsig_e				
Constant	-0.400***	-0.388***	-0.327***	-0.535***
Observations	9406	10628	11597	11608

Table S.6: Robustness Analysis – Excluding Dementia Diagnoses (Linear Time Trend)

	(1) Orientation	(2) Numeracy	(3) Memory	(4) Verbal Fluency
East	0.034	0.010	0.035	0.021
Time trend	-0.012***	-0.019***	-0.107***	-0.026***
East * Time trend	0.012**	0.022***	0.089***	0.021***
Age at baseline	-0.033***	-0.032***	-0.026***	-0.015***
Number of books (age 10)	0.069***	0.087***	0.015*	0.036***
Math skills (relative to others, age 10)	-0.080***	-0.067***	-0.036***	-0.144***
Language skills (relative to others, age 10)	-0.074***	-0.071***	-0.008	-0.020
Childhood health (up to age 15): Excellent				
Very good	0.008	-0.015	-0.004	0.028
Good	-0.040	-0.066*	-0.016	-0.004
Fair	-0.058	-0.108**	-0.029	-0.021
Poor	-0.091	-0.046	-0.051	-0.070
Varied a lot	0.136	-0.010	0.008	-0.037
Birth cohort: born before 1934	0.000	0.000	0.000	0.000
born between 1934 and 1945	0.150***	0.123**	-0.038	0.056
born between 1946 and 1952	0.103*	-0.030	-0.768***	-0.232***
born between 1953 and 1959	0.065	0.049	-0.344***	-0.082
born after 1959	0.019	-0.042	-0.500***	-0.168**
Gender: Woman	0.182***	0.067**	-0.040*	-0.268***
Education: low	0.000	0.000	0.000	0.000
Education: intermediate	0.305***	0.268***	0.049	0.353***
Education: high	0.538***	0.519***	0.092**	0.440***
Lives in urban area	0.022	-0.035	0.016	0.019
Lives with partner or spouse	0.018	0.059*	0.106***	0.002
Size of household	-0.011	-0.034*	-0.034*	-0.026
Income: Lowest quintile	0.000	0.000	0.000	0.000
Second quintile	-0.030	0.088***	0.005	0.153***
Third quintile	0.034	0.151***	-0.001	0.184***
Highest quintile	0.047*	0.192***	-0.032	0.189***
Lives in urban area	0.079***	0.062**	0.019	0.093***
Constant	5.134***	4.763***	9.217***	5.052***
lns1_1_1				
Constant	-4.104***	-3.588***	-2.802***	-4.628***
lns1_1_2				
Constant	-0.737***	-0.485***	-1.480***	-0.572***
lnsig_e				
Constant	-0.351***	-0.534***	-0.287***	-0.374***
Observations	16966	14335	12087	13359

* $p < 0.05$, ** $p < 0.01$, *** $p < 0.001$
